# Nutrient Composition and Health Information on the Labels of Commercially Produced Complementary Foods in Nepal

**DOI:** 10.1111/mcn.70150

**Published:** 2026-03-15

**Authors:** Nisha Sharma, Prabhat Lamichhane, Penelope Love, Pradeep Kaji Poudel, Colin Bell

**Affiliations:** ^1^ School of Medicine, Faculty of Health Deakin University Geelong Victoria Australia; ^2^ Department of Public Health School of Psychology and Public Health La Trobe University Melbourne Victoria Australia; ^3^ School of Exercise and Nutrition Sciences Faculty of Health Deakin University Geelong Victoria Australia; ^4^ Department of Food Technology Golden Gate International College Tribhuvan University Kathmandu Nepal

**Keywords:** commercially produced complementary foods, food labelling, infants, Nepal, nutrient composition, young children

## Abstract

Commercially produced complementary foods (CPCFs) – commercially produced food or beverage products that are specifically marketed as suitable for feeding infant and young children (IYC) from 6‐36 months of age – are increasingly available in Southeast Asia and purchased by caregivers. However, we know little about the nutrient composition of these foods or health‐related information on the packaging of the CPCFs available in Nepal. This study assessed nutrient composition and health information on the labels of CPCFs available in urban Nepal. In 2024, we visited 4 large stores (2 supermarkets and 2 departmental stores), 22 small stores (corner stores), and 2 baby stores in Kathmandu Valley. We photographed all CPCFs. To analyse, we extracted nutrient and health information from the label and categorized each CPCF into WHO Southeast Asia Region Nutrient Profile and Promotion model (SEAR NPPM) food sub‐categories. Within each category we calculated the proportion meeting energy and nutrient thresholds for nutrient of concerns (protein, total sugar, sodium, and total fat threshold per 100g of product) from the SEAR NPPM and the proportion with health‐related labels that complied with SEAR NPPM labelling requirement. We found 61 CPCFs available in seven (3 large, 2 small and 2 baby stores) out of the 28 stores visited. None of these CPCFs met all energy and nutrient thresholds, 75.6% met energy thresholds, 79.5% total fat content thresholds, 50.8% sodium content, and 25% total sugar content. Total sugar content was double the SEAR NPPM threshold of ≤ 3g/100 g, ranging from 1g/100 g for CPCFs in the ‘cereals without added high protein’ sub‐category to 27.4g/100 g for CPCFs in rusks and biscuits’ sub‐category. Less than five (3.3%) of assessed CPCFs were compliant with the SEAR NPPM labelling requirement. CPCFs in Nepal contained high levels of nutrients of concern and had misleading health information on the labels. To safeguard health and nutrition of older infants and young children, Nepal has a mandatory food standard for cereal‐based commercial complementary food that determines nutrient composition, promotional requirements, and food safety parameters. In addition to stronger enforcement of this standard, we recommend similar standards be applied for other CPCF sub‐categories.

## Summary

- None of the commercially produced complementary foods (CPCFs) available in Nepali market met all energy and nutrient thresholds; sugar and sodium content for more than half CPCFs were above the thresholds.
- Only 3.3% of assessed CPCFs were compliant with the labelling requirement, with some products targeting infants under 6 months of age and undermining breastfeeding.
- It is important that the national standards for CPCFs are enhanced and enforced to safeguard nutrition and health of Nepali infant and young children.

## 1 Introduction

The first 2 years of a child's growth and development are an opportunity for parents/carers to help them establish healthy food preferences (Mennella et al. 2016). To achieve optimal nutrition over this period, WHO recommends exclusive breastfeeding from birth to 6 months of age, and from 6 months, introducing complementary foods and on‐demand breastfeeding up to 2 years of age and beyond (World Health Organization 2023). However, infant and young child (IYC) feeding practices are suboptimal in Nepal with only 48% of children aged 6–23 months receiving minimum acceptable diets—diets with minimum diversity (containing at least five of eight food groups in a day) in age‐appropriate minimum frequency (receiving meals at least three or four times in a day) (Ministry of Health and Population MoHP, New Era, & ICF International 2017). Undernutrition is persistent, with stunting at 25% and underweight at 19% (Ministry of Health and Population MoHP, New Era, & ICF International 2017).

Achieving nutrition quality thresholds in complementary foods is of paramount importance for the optimal growth and development of Nepali IYC. The 2016 WHO's ‘Guidance on Ending the Inappropriate Promotion of Foods for Infants and Young Children’ recommends the use of a nutrient profile model (NPM) for defining these thresholds and guiding decisions on the appropriateness of claims so that foods high in energy and/or sodium, total sugar, or total fat are not promoted (WHO 2017). Nutrient Profile and promotion Models (NPPMs) for IYC have subsequently been developed for Europe and Southeast Asia to assess the nutrient composition and labelling practices of commercial foods marketed as suitable for children 6–36 months of age (WHO Regional Office for Europe 2022; WHO South‐East Asia Region 2023).

Commercially produced complementary foods (CPCFs) are defined by WHO as ‘commercially produced food or beverage products that are specifically marketed as suitable for feeding IYC from 6 to 36 months of age’ (WHO 2017). CPCFs are increasingly available and frequently purchased by parents and caregivers in high and low‐income countries (Debessa et al. 2023; Foterek et al. 2015; McCann et al. 2022; Walls et al. 2023). In many countries and regions including Australia, the UK, India, and Southeast Asia, parents and caregivers perceive these products as convenient, healthy, nutritious, and safe for their children (Isaacs et al. 2022; McCann et al. 2022; Singh 2024; Walls et al. 2023). However, studies of IYC from Australia and US have reported that more than 60% of CPCFs do not meet WHO thresholds for energy, sodium, total sugar, added sugar, total fat, and fruit content (Coyle et al. 2024; Dunford et al. 2024). Non‐compliance with WHO recommendations was also observed in Africa and Southeast Asia, where approximately 85% of CPCFs did not comply (Khosravi et al. 2023; Pries et al. 2023). Moreover, inappropriate promotion on the labels of CPCFs, which idealised the product, was observed in all these countries.

With evidence suggesting CPCFs are increasingly available in Southeast Asia (Blankenship et al. 2023), there is scant evidence on CPCF availability, nutrient composition and promotion in Nepal. A 2016 cross‐sectional study that scoped 31 large and small retail stores in Kathmandu found 22 CPCFs in the stores (Sweet et al. 2016), but there is no evidence on the proportion of CPCFs that meet the nutrient and health‐related labelling requirements of the WHO Southeast Asia Region Nutrient Profile and Promotion Model (SEAR NPPM). This study aims to fill this gap by describing nutrient composition and health information on the labels of CPCFs available in urban Nepal.

## 2 Methods

### 2.1 Study Design and Setting

We conducted a cross‐sectional study of packaged food products, including CPCFs, available in retail stores in Kathmandu Valley in February–April 2024. We chose Kathmandu Valley because it is the largest city in Nepal and likely to have the highest availability of packaged food products. We obtained a letter of support from the Department of Food Technology and Quality Control, Ministry of Agriculture and Livestock Development for data collection and invited 5 large stores (supermarket and departmental store), and 30 small stores (corner stores) in Kathmandu Valley (see Sharma et al. 2025 for detailed methods). Additionally, Kathmandu Valley had two stores that exclusively sold baby foods (hereafter baby stores) that were also invited to participate. We sought verbal consent from the store owner/manager to photograph products, and if they did not provide consent, we purposively selected another large store on our list or visited another small store nearby (50 m). Ethics was not required for this study as we collected publicly available food label information.

### 2.2 Product Identification and Data Collection

Based on WHO criteria (WHO 2017a), we identified and collected data from all CPCFs in the stores that featured one or more of the following on the label: (1) an age recommendation for children aged 6–36 months; (2) a label containing words like baby, infants, toddler; (3) image(s) of a 36 month (or younger) child or a child feeding with a bottle; (4) presentation as suitable for children 36 months and under (e.g., statement around use or benefit of the product for children under 36 months of age, presence of image of bottle or statement suggesting use of bottle for feeding). Unique CPCFs were defined based on brand, sub‐brand, manufacturer, country of manufacture, and age category specified on the label. All unique CPCFs available in the stores were photographed. NS provided 1‐day training, including the first half of classroom training and the second half of field practice, to local research assistants (SP, BG) to identify and photograph CPCFs and record product information. CPCFs were photographed in‐store from all sides to ensure all information on the label was captured, including name, brand, manufacturer, nutrition information, ingredient list, nutrition claims, and statements. At the end of each day, NS, SP and BG entered product name, brand, recommended age (in months), manufacturer name and country of manufacture, and unique photo identification numbers for each CPCF in a data entry form created in Qualtrics XM. Photographs with a unique photo identification number were then transferred via NS's computer to a secure Deakin University server.

### 2.3 Data Extraction and Processing

#### 2.3.1 Assessing Compliance With Nutrient Thresholds

We exported information from Qualtrics to Microsoft Excel to determine nutrient and labelling compliance of CPCFs. NS trained SP and BG to extract information from the images of the label and moderated the process. SP and BG extracted information on serving size, ingredient list, energy, protein, total sugar, total fat, and sodium for each CPCF from the photographs of labels into Microsoft Excel. We used the SEAR NPPM to determine nutrient thresholds. SEAR NPPM was chosen because it is tailored to the region and is designed to assess both nutritional quality (aligning with Codex standards for baby foods) and promotional strategies (aligning with WHO's recommendations on ending inappropriate promotion of foods for infants and young children) of CPCFs. NS classified each CPCF into SEAR NPPM food categories and sub‐categories. Refer to Table 1 for descriptions and examples of SEAR NPPM's food sub‐categories.

**Table 1:** Description of category and sub‐category of commercially produced complementary foods (CPCF) from WHO SEAR NPPM (WHO South‐East Asia Region 2023).

Category	Sub‐category	Description	Example
Cereal‐based processed foods	Cereals without added high proteins	Cereals that are or have to be prepared for consumption with milk or other appropriate nutritious liquids.	Cereal porridge mix, pancake mix, millet powder, dry pseudo‐cereal (such as quinoa, amaranth, chia, buckwheat) powder.
	Cereals with added high‐protein foods	Cereals with an added high protein food that are or have to be prepared for consumption with water or other appropriate protein‐free liquid.	Cereal‐based (such as cereal– milk/cereal–vegetable or fruit/cereal–pulses) porridge mix, cereal‐based health‐drink, oats, multigrain mix, cereal‐based bars.
	Pasta, noodles, and like products	Pasta, noodles, and like products that are to be used after cooking in boiling water or other liquids.	Pasta, noodles, spaghetti, macaroni, vermicelli.
	Rusks and biscuits	Rusks and biscuits that are to be used either directly or, after the addition of water, milk, or suitable liquid.	Rusks, crackers, biscuits, cookies, puffs/melts, sticks, crunchies, crisps, wafers.
Canned baby foods	Legumes and pulse‐based foods	Chickpeas, lentils, peas, green gram, kidney beans, and soya beans.	Porridge (such as green gram porridge).
	Products with animal protein source as the only ingredient	Meat, poultry, fish, or other source of protein are the only ingredients mentioned in the name of the product.	Canned meat/chicken/fish, chicken nuggets, fish fingers, chicken patty, steamed meat/chicken/fish.
	Products with animal protein source in single or in combination	Products with animal protein sources singularly or in combination, and mentioned first in the name.	Creamed porridge, yoghurt, chicken/meat risotto, beef or chicken sauce (for pasta‐like products), beef puree, chicken‐based products.
	Dairy foods	Dishes with dairy products as the first or only ingredient.	Milk‐based smoothies, yogurt, custard, pudding.
	Fruit and vegetable‐based products	Products containing fruits and vegetables as the first or the only ingredient in the name.	Fruits: fruit puree, fruit smoothies, freeze‐dried fruit, fruit crisps, fruit‐based desserts. Vegetables: vegetable puree, vegetable soups, vegetable juices, vegetable chips.

NS double‐entered 5% data to ensure the accuracy of extracted nutrient data. Cohen's Kappa was used to assess inter‐rater agreement for each nutrient. A strong and significant (*p* < 0.000) agreement was found for energy (*κ* = 0.87), protein (*κ* = 1.00), total fat (*κ* = 1.00), and total sugar (*κ* = 0.85). A moderate agreement (*κ* = 0.59) was found for sodium. All sodium values were reviewed against the label, and inconsistencies were rectified. For CPCFs that needed reconstitution before consumption, we calculated reconstituted nutrient values using the manufacturer's instructions. CPCFs with specific nutrient information missing were considered not meeting that nutrient threshold.

#### 2.3.2 Assessing Compliance With Labelling Requirements

NS assessed labelling compliance by recording ‘Yes’ or ‘No’ in the spreadsheet in response to the 13 SEAR NPPM labelling requirements (see Table 2 for description and examples of labelling requirements). CB double‐entered responses assessing 5% of the products against the labelling requirements. Agreement of more than 85% was found between the coders for the labelling requirement variables, except for ‘no statement relating to convenience or lifestyle’ (75% agreement) and ‘no presence of statement conveying ideal for optimum feeding or superior to home‐made foods with local ingredients’ (62% agreement). Inconsistencies in these criteria were resolved through discussion, and the database was updated.

**Table 2:** WHO SEAR NPPM labelling requirements: description and examples.

Labelling requirement	Details and examples
*A. Protection and promotion of breastfeeding*	
1. Presence of a statement on the suitable age of introduction as 6 months or more	The suitable age of introduction of food products, as more than or equal to 6 months, must be stated
2. No presence of image/text/other to indicate or promote the use of a product for infants under 6 months of age	No image/text/other should indicate/promote the use of products for infants < 6 months of age, including through references to milestones and/or development stages.
3. Presence of a statement on the importance of continued breastfeeding for 2 years and beyond	A statement on the importance of continued breastfeeding for 2 years and beyond must be stated.
4. No presence of image/text suggesting equivalence or superiority to breast milk	No image/text should be presented to undermine breastfeeding or suggest equivalence or superiority to breastmilk.
*B. No nutrition and health claims*	
5. No nutrition claim	Nutrition claims such as nutrient content, nutrient comparative, and nutrient function claims are not permitted on packaging. Examples include: ‘Nutritionally balanced’, ‘provides good nutrition to children’, ‘a source of…’ [minerals, vitamins, iron, dietary fibre, omega‐3, probiotics, prebiotics, protein, amino acids, DHA, etc.]
6. No health claim	Health claims, such as other function claims or reduction of disease risk, are not permitted on packaging. Examples include: ‘Good for…’, ‘improves…’, ‘needed for…’ [healthy growth, development, digestion, appetite, learning to chew, bones and teeth, the brain, eyes, preventing iron deficiency anaemia, cognitive development, immune system]
7. No non‐addition claim	No presence of a statement on the non‐addition of ingredients generally perceived to be harmful. Non‐addition claims are not permitted on packaging.
	Examples include: ‘no added…’, ‘low in…’, ‘no.’ [sugar, salt, artificial flavour/colour, additives/preservatives, etc.]
*C. No statements to idealise the product*	
8. No presence of statement relating to ideal taste	A statement that idealises the taste of the product is not permitted. Examples include: ‘delight for tiny taste buds’, ‘tasty/yummy/delicious’, ‘whole family loves it’
9. No presence of statement conveying ideals on optimum feeding or superior to home‐made foods with local ingredients	Statements that idealise products in any way, convey ideals on optimum feeding, or imply that products are superior to suitable homemade foods with local ingredients or family food are not permitted. Examples include: ‘ideal for tiny tummies’, ‘helps lay foundations for toddler's future progress’, ‘making the right feeding choices for your baby’, ‘carefully prepared by our baby food experts’, ‘perfect/ideal way to introduce foods’
10. No presence of statement relating to convenience or lifestyle	Statements relating to convenience or lifestyle are not permitted. Examples include: ‘ideal for the meals on go’, ‘simply to top up between meals’, ‘convenient’, ‘great for a busy and active life’
11. No presence of a statement implying a particular standard	Characteristic‐based statements that imply a relationship with a particular standard and include words like ‘pure’, ‘fresh’, ‘organic’, ‘genuine’, ‘ideal’, and ‘natural’ are not permitted. Examples include: ‘100% natural’, ‘real/fruit vegetables’, and ‘Only use of specially selected ingredients’.
*D. Statements to include*	
12. Presence of a statement discouraging caregivers from allowing IYC to suck food directly via spout (Applicable to soft ready‐to‐eat products like puree)	Ready‐to‐eat pureed foods sold in packs with a spout must include a statement to discourage caregivers from allowing infants and young children to suck the food directly via the spout. Examples include: ‘IYC should not be allowed to suck directly from the pouch/container’
13. Presence of preparation instructions (Applicable to products that need preparation before consumption)	Products that need preparation before consumption should include preparation instructions.

### 2.4 Data Analysis

We used Stata 18.0 to describe the data. We calculated the number and proportion of CPCFs that met SEAR NPPM's energy and nutrient thresholds per 100 grams (g) of product for each food subcategory (WHO South‐East Asia Region 2023). The WHO energy threshold for all food groups except ‘fruit and vegetable‐based products’ is ≥ 80 kcal/100 g. The protein threshold is 2.4–4.4 g/100 g for ‘cereals with added high protein food’ and ‘rusks and biscuits’. For ‘dairy foods’, the protein threshold is ≥ 1.76 g/100 g. Total fat threshold for ‘cereals without added high protein’ and ‘rusks and biscuits’ is ≤ 2.64 g/100 g, and for ‘cereals with added high protein food’ and ‘dairy foods’ is ≤ 3.6 g/100 g. Total sugar threshold for all food groups is ≤ 3 g/100 g, and sodium threshold for all food groups is ≤ 0.04 g/100 g. We determined the number and proportion of CPCFs meeting the SEAR NPPM 13 labelling requirements described in Table 2. We report the median value and IQR of energy and nutrients of concern.

## 3 Results

We approached 37 retail stores for data collection. Nine stores declined participation—one large store (1/5, 80.0% response rate) and eight small stores (8/30, 73.3% response rate). From the participating 28 stores, CPCFs were identified in seven stores (3/4 large, 2/22 small, and 2/2 baby stores) indicating that small corner stores were not a major source of CPCFs.

Sixty‐one unique CPCFs were identified within five of the nine SEAR NPPM food sub‐categories, namely, ‘cereals without added high protein’, ‘cereals with added high protein’, ‘rusks and biscuits’, ‘fruit and vegetable‐based products’, and ‘dairy foods’. Of these, a third were ‘rusks and biscuits’ (32.8%, *n* = 20) and ‘cereals with added high proteins’ (31.1%, *n* = 19) (Table 3). Only nine CPCFs (14.8%) were manufactured in Nepal. Others were imported from Australia (34.4%, *n* = 21), Malaysia (27.9%, *n* = 17), India (11.5%, *n* = 7), USA (8.2%, *n* = 5), Spain (1.6%, *n* = 1), and Italy (1.6%, *n* = 1) (data not shown).

**Table 3:** Number and percentage of commercial complementary foods meeting WHO SEAR NPPM threshold for energy, protein, total sugar, total fat, and sodium.

Category (*n*, %)	Sub‐category (*n*, %)	Energy *n* (%)	Protein *n* (%)	Total fat *n* (%)	Total sugar *n* (%)	Sodium *n* (%)	Met overall composition *n* (%)
Cereal‐based processed foods (*n* = 47, 77.0%)	Cereals without added high proteins (*n* = 8, 13.1%)	0 (0.0%)	—	8 (100.0%)	7 (87.5%)	8 (100.0%)	0 (0.0%)
	Cereals with added high protein foods (*n* = 19, 31.1%)	16 (84.2%)	10 (52.6%)	18 (94.7%)	4 (21.1%)	10 (52.6%)	0 (0.0%)
	Rusks and biscuits (*n* = 20, 32.8%)	20 (100.0%)	1 (5.0%)	12 (60.0%)	1 (5.0%)	11 (55.0%)	0 (0.0%)
Canned baby foods (*n* = 14, 23.0%)	Dairy foods (*n* = 2, 3.3%)	2 (100.0%)	2 (100.0%)	1 (50.0%)	0 (0.0%)	2 (100.0%)	0 (0.0%)
	Fruit and vegetable‐based products (*n* = 12, 19.7%)	—	—	—	0 (0.0%)	0 (0.0%)	0 (0.0%)
	All products (61)	34 (75.6%)	13 (32.5%)	39 (79.6%)	12 (19.7%)	31 (50.8%)	0 (0.0%)

(−) denotes no threshold provided for the food groups in SEAR NPPM.

### 3.1 Nutrient Composition

None of the CPCFs met all SEAR NPPM nutrient composition thresholds (Table 3). Approximately three‐quarters met individual energy and total fat thresholds, 75.6% (*n* = 34) and 79.6% (*n* = 39) respectively. Half (50.8%, *n* = 31) met the sodium threshold, a third (32.5%, *n* = 13) met the protein threshold and less than a quarter (19.7%, *n* = 12) met the total sugar threshold. Overall, the median content of energy and nutrients of concern, except total sugar, in CPCFs was within the required range of SEAR NPPM (Table 4). Median total sugar content was double the WHO threshold of ≤ 3.0 g/100 g, ranging from 1.0 g/100 g for CPCFs in the ‘cereals without added high protein’ sub‐category to 27.4 g/100 g for CPCFs in ‘rusks and biscuits’ sub‐category.

**Table 4:** Median energy, total fat, total sugar, and sodium content per 100 grams of commercial complementary food^a^.

Food group	Food sub‐group	Energy per 100g^b^		Protein per 100g^c^		Total fat per 100 g d		Total sugar per 100g^e^		Sodium per 100 g^f^	
		*N*	Median (IQR)	*N*	Median (IQR)	*N*	Median (IQR)	*N*	Median (IQR)	*N*	Median (IQR)
Cereal‐based processed foods (*n* = 47, 77.0%)	Cereals without added high proteins (*n* = 8, 13.1%)	8	44.40 (41.80–44.70)	8	1.05 (0.70–1.30)	8	0.70 (0.60–0.90)	8	1. 00 (0.70–1.80)	8	0.00 (0.00–0.00)
	Cereals with added high protein foods (*n* = 19, 31.1%)	15	108.70 (102.20–114.40)	18	4.00 (3.70–4.40)	18	2.30 (1.30–2.40)	12	5.90 (1.50–6.10)	10	0.03 (0.00–0.030)
	Rusks and biscuits (*n* = 20, 32.8%)	20	386.00 (379.50–398.00)	20	7.35 (6.70–7.70)	20	1.55 (0.95–7.50)	14	27.40 (12.10–37.20)	17	0.01 (0.01–0.10)
Canned baby foods (*n* = 14, 23.0%)	Dairy foods (*n* = 2, 3.3%)	2	95.30 (95.00–95.60)	2	2.35 (2.00–2.70)	2	3.30 (2.90–3.70)	2	10.10 (9.20–11.00)	2	0.03 (0.02–0.04)
	Fruit and vegetable‐based products (*n* = 12, 19.7%)	12	65.0 (57.40–77.10)	12	0.60 (0.20–1.35)	12	0.65 (0.20–1.35)	12	8.10 (5.90–9.80)	12	0.00 (0.00–0.01)
	All products (61)	57	103.50 (60.00‐380.00)	60	3.75 (1.30–6.80)	60	1.30 (0.80–2.40)	48	6.40 (2.80–11.50)	49	0.01 (0.01–0.03)

^a^ Products with missing specific nutrient content are not included.

^b^ Energy threshold for all food groups except fruit and vegetable‐based products: ≥ 80.00 kcal/100 g.

^c^ Protein threshold for cereals with added high protein food and rusks and biscuits: 2.40‐4.40 g/100 g; for dairy foods: ≥ 1.76 g/100g.

^d^ Total fat threshold for cereals without added high protein and rusks and biscuits: ≤ 2.64 g/100 g; for cereals with added high protein food and dairy foods: ≤ 3.60 g/100 g.

^e^ Total sugar threshold for all food groups: ≤ 3.00 g/100g.

^f^ Sodium threshold for all food groups: ≤ 0.04 g/100 g.

All products in the ‘cereals without added high protein’ sub‐category met the total fat (100.0%, *n* = 8) and sodium (100.0%, *n* = 8) thresholds, and most met the total sugar (87.5%, *n* = 7) threshold. Interestingly, only 52.6% (*n* = 2) of CPCFs in the ‘cereals with added high protein’ sub‐category met the protein threshold, compliance with total sugar and sodium threshold was also low for this sub‐category at 21.1% (*n* = 4) and 52.6% (*n* = 10) respectively. Only 5.0% (*n* = 1) of CPCFs in the ‘rusks and biscuits’ sub‐category met protein and total sugar thresholds. All CPCFs in the ‘dairy foods’ sub‐category met energy, protein, and sodium threshold, but none met the total sugar threshold. No CPCFs in the ‘fruit and vegetable‐based products’ sub‐category met thresholds for total sugar or sodium.

### 3.2 Labelling Requirement

The proportion of CPCFs that complied with all SEAR NPPM labelling requirements was low at 3.3% (*n* = 2) (Table 5); only two CPCFs (in the ‘rusks and biscuits’ sub‐category) met all 13 WHO labelling requirements.

**Table 5:** Number and percentage of commercial complementary foods complying with SEAR NPPM labelling requirements, *n* (%).

Labelling requirement	Overall product (*n* = 61)	Cereals without added high proteins (*n* = 8)	Cereals with added high protein (*n* = 19)	Rusks and biscuits (*n* = 20)	Dairy foods (*n* = 2)	F&V based product (*n* = 12)
**Protection and promotion of breastfeeding**						
States suitable age of introduction of food products as 6 months or more	52 (85.3%)	5 (62.5%)	16 (84.2%)	20 (100.0%)	2 (100.0%)	9 (75.0%)
No presence of image/text/other to indicate or promote use of food product for infants under 6 months of age	52 (85.3%)	5 (62.5%)	16 (84.2%)	20 (100.0%)	2 (100.0%)	9 (75.0%)
States the importance of continued breastfeeding for two years and beyond	15 (24.6%)	1 (12.5%)	1 (5.3%)	13 (65.0%)	0 (0.0%)	0 (0.0%)
No presence of image/text suggesting equivalence or superiority to breastmilk	61 (100.0%)	8 (100.0%)	19 (100.0%)	20 (100.0%)	2 (100.0%)	12 (100.0%)
Compliance with “protection and promotion of breastfeeding”	**15 (24.6%)**	**1 (12.5%)**	**1 (5.3%)**	**13 (65%)**	**0 (0%)**	**0 (0%)**
**Nutrition and health claims**						
No nutrition claim	21 (34.4%)	0 (0.0%)	3 (15.8%)	11 (55.0%)	1 (50.0%)	6 (50.0%)
No health claim	33 (54.1%)	5 (62.5%)	7 (36.8%)	9 (45.0%)	2 (100.0%)	10 (83.3%)
No non‐addition claim	17 (27.9%)	0 (0.0%)	12 (63.2%)	5 (25.0%)	0 (0.0%)	0 (0.0%)
Compliance with “no nutrition and health claims”	**4 (6.6%)**	**0 (0.0%)**	**2 (10.5%)**	**2 (10.0%)**	**0 (0.0%)**	**0 (0.0%)**
**No Statements that idealise the product**						
No presence of a statement relating to ideal taste	37 (60.7%)	5 (62.5%)	14 (73.7%)	15 (75.0%)	1 (50.0%)	2 (16.7%)
No presence of a statement conveying ideals on optimum feeding or superior to home‐made foods with local ingredients	26 (42.6%)	2 (25.0%)	5 (26.3%)	14 (70.0%)	2 (100.0%)	3 (25.0%)
No presence of a statement relating to convenience or lifestyle	49 (80.3%)	7 (87.5%)	18 (94.7%)	16 (80.0%)	2 (100.0%)	6 (50.0%)
No presence of a statement implying standard	34 (55.7%)	0 (0.0%)	16 (84.2%)	11 (55.0%)	2 (100.0%)	5 (41.7%)
Compliance with “no statements that idealise the product”	**13 (21.3%)**	**0 (0.0%)**	**5 (26.3%)**	**7 (35.0%)**	**1 (50.0%)**	**0 (0.0%)**
**Statements to include**						
Discouraging to suck food directly via spout (Applicable to soft ready to eat products)	12 (85.7%)	NA	NA	NA	1 (50.0%)	11 (91.7%)
Preparation instruction (Applicable to cereal‐based products requiring preparation)	24 (88.9%)	8 (100.0%)	16 (84.2%)	NA	NA	NA
**Overall compliance**	**2 (3.3%)**	**0 (0.0%)**	**0 (0.0%)**	**2 (10.0%)**	**0 (0.0%)**	**0 (0.0%)**

*Note:* Bold values indicate compliance of sub‐categories.

Overall, a quarter of CPCF labels (24.6%, *n* = 15) complied with the four labelling requirements for the “protection and promotion of breastfeeding”, with prevalence ranging from 0.0% (*n* = 0) for ‘dairy foods’ and ‘fruit and vegetable‐based products’ sub‐categories to 65.0% (*n* = 13) for ‘rusks and biscuits’ sub‐category. Most labels (85.3%, *n* = 52) recommended the suitable age of introduction of CPCFs as 6 months or older. However, 14.7% (*n* = 9) of products had labels that indicated they were suitable for children under 6 months of age. Only a quarter of CPCFs (24.6%, *n* = 15) included a statement on the importance of continued breastfeeding for 2 years and beyond. No CPCFs (100.0%, *n* = 61) claimed that the products were equivalent or superior to breastfeeding.

Overall compliance with the three labelling requirements for ‘no nutrition and health claims’ was 6.6% (*n* = 4). More than a third (34.4%, *n* = 21) of CPCFs had no nutrition claims, with the ‘cereals without added high protein’ sub‐category (0.0%, *n* = 0) and ‘cereals with added high protein food’ sub‐category (15.8%, *n* = 3) being least likely to have no nutrition claims. More than half of the CPCFs (54.1%, *n* = 33) had no health claims with the ‘cereals with added high protein food’ subcategory (36.8%) being least likely to have no health claims. Only 27.9% (*n* = 17) CPCFs complied with the no non‐addition claim, with zero compliance in the sub‐categories of ‘cereals without added high proteins’, ‘dairy foods’, and ‘fruit and vegetable‐based products’.

Overall, 21.3% of CPCFs' labels complied with the four labelling requirements for ‘no statement to idealise product’, with no CPCFs in ‘cereals without added high protein’ and ‘fruit and vegetable‐based products’ sub‐categories complying with the requirements. While 80.3% (*n* = 49) of labels did not have statements relating to convenience or lifestyle, only 42.6% (*n* = 26) did not have statements conveying ideals on optimum feeding or superior to home‐made foods with local ingredients. Over half of CPCFs (55.6%, *n* = 11) did not have statements implying meeting nutrition standards.

For ‘statements to include’, 91.7% (*n* = 11) of labels in the ‘fruit and vegetable‐based products’ sub‐categories, had a statement ‘discouraging caregivers from allowing IYC to suck food directly via spout’ but only 1 of the 2 ‘dairy foods’ did. For the two cereal‐based products sub‐categories, there were some labels (15.8%, *n* = 3) in the ‘cereals with added high protein’ sub‐category that did not have adequate preparation instructions.

## 4 Discussion

This cross‐sectional audit of CPCFs in 28 retail stores in Kathmandu Valley found 61 unique CPCFs in seven stores, mostly large stores and specialist baby stores. Of these products, most were cereal‐based (77.0%) and only 14.6% were manufactured in Nepal. No CPCFs met all SEAR NPPM's energy and nutrient thresholds. Approximately half of the products met the sodium threshold, a third met the protein threshold, and less than a quarter met the total sugar threshold. Only 3.3% of products complied with all SEAR NPPM labelling requirements. A quarter of labels complied with labelling requirements to ‘protect and promote breastfeeding’, 6.6% with ‘no nutrition and health claims’, and 21.3% with ‘no statements to idealise the product’. A small number of products did not have adequate preparation instructions.

A 2016 survey conducted in 31 large and small stores (supermarkets, corner stores, pharmacies) of Kathmandu Valley in 2013 found 22 CPCFs, of which 21 were infant cereals and one was puree (Sweet et al. 2016). Our study, 11 years later, adds to the literature, showing that the number and variety of CPCFs available in stores have increased, notably rusks and biscuits, dairy foods (e.g. yoghurts), and fruit‐ and vegetable‐based products (e.g. puree). In contrast to markets in countries like Australia and US (Coyle et al. 2024; Dunford et al. 2024), other canned baby foods based on legumes, pulses or animal proteins were not observed in Nepali stores. This is likely because CPCF market is still growing in Nepal, and high availability of range of CPCFs in more developed countries (Bassetti et al. 2023; Coyle et al. 2024; Dunford et al. 2024; Hutchinson et al. 2021) suggests that the number and variety of CPCFs are likely to increase over time in Nepal. Other Southeast Asian countries already have high availability of CPCFs (Bassetti et al. 2023; Blankenship et al. 2023; Hinnouho et al. 2023), and there has been a rapid increase in other commercial baby foods, such as milk formula, in South Asia (Baker et al. 2021). Several factors are likely to be driving the supply and demand of these products including global expansion of baby food manufacturing and supply chains, originated in high income countries and proliferating in low and middle income countries (Baker et al. 2021), and economic growth and rapid urbanisation, including in Nepal (Subedi et al. 2017). Also, with the gradual increase in workforce participation of women in the formal sector (27.6% as of 2023) in Nepal (World Bank n.d.), the convenience of CPCFs (Isaacs et al. 2022; Singh 2024; Walls et al. 2023) is likely to increase demand. In Ethiopia, a similar low‐ and middle‐income setting to Nepal, a cross‐sectional study found that maternal employment was significantly associated with the use of commercial complementary feeding (Debessa et al. 2023).

Our study findings reflect broader global concerns regarding the low nutritional quality of baby foods. Studies from Australia and US reported less than 40% of baby foods meet the WHO (Euro) nutrient composition requirement, and a multi‐country study in Southeast Asia found less than 15% of baby foods met the nutrient composition requirement. CPCFs exceeding the total sugar threshold for IYC in our study were substantially higher (80.3%) than levels reported in these countries (44.6% in US, 38.0% in Australia, and > 50% in the Southeast Asian countries) and all studies, ours included, identified ‘rusks and biscuits’ as the sub‐category of most concern. (Bassetti et al. 2022; Coyle et al. 2024; Dunford et al. 2024; Pries et al. 2023). Median total sugar content for this category was 27.40 g per 100 g, higher than that of 21.90 g per 100 g we observed for general bakery products in an earlier study (Sharma et al. 2025). A qualitative study has reported that Nepali caregivers of children 12–23 months, specifically those in paid employment, considered packaged foods, such as biscuits, the most convenient foods to feed young children (Sharma et al. 2019). This demand for convenience may expose Nepali IYC to more sugar through CPCFs than the general population through bakery products. Also, nearly half of the CPCFs in our study contained excess sodium, consistent with the Southeast Asian study on the sub‐category ‘finger foods and snacks’ for IYC, where more than half of products exceeded the sodium threshold (< 0.05 g per 100 g) (Pries et al. 2023).

Although there is a lack of national data on sugar and sodium intake among Nepali infants and young children, an earlier study in Kathmandu Valley by Pries et al. (2019) found that total energy, sugar and sodium intake from unhealthy snack foods and beverages among children aged 12–23 months in Kathmandu Valley was 24.5%, 31.1% and 44.9% respectively. Considering these findings together with the nutrient composition of CPCFs in our analysis, there is a potential risk of overweight and obesity and NCDs to Nepali IYC from dietary exposure to nutrient poor food sources. In addition to concerns about high levels of specific nutrients of concern in CPCFs, studies have reported that CPCFs displace fruit and vegetable intakes (Foterek et al. 2015), reducing micronutrient‐rich foods in the diets of IYC. We did not measure consumption in our study but if displacement occurs in Nepal, where intake of fresh fruit and vegetables is already low, undernutrition and micronutrient deficiencies will persist (MoHP et al. 2022).

Promoting exclusive breastfeeding for the first 6 months of life is a national and global priority (Karn et al. 2017; WHO 2017). In our study, only a quarter of product labels complied with the WHO recommendation to protect and promote breastfeeding for 2 years and more of a child's life (WHO 2017). This finding is consistent with observations from seven Southeast Asian countries using the WHO NPPM for the European region (Pries et al. 2023). Our study found that 15.0% of CPCFs were being promoted for children under 6 months of age, which is an increase from 13.6% reported by an earlier study conducted in Kathmandu Valley (Sweet et al. 2016). A qualitative study of mothers in the UK by Isaacs et al. (2022) reported that stating a recommended age of 4 or 5 months on a product label encouraged mothers to start solids at an early age, illustrating how such labelling can undermine exclusive breastfeeding for infants under 6 months of age.

According to WHO, labels should not idealise CPCFs by making nutrition, health and other claims (WHO 2017). However, we found many product labels included such claims and this is consistent with the findings from studies in Asia, Australia, Africa, and the United States of America (Bassetti et al. 2023; Coyle et al. 2024; Dunford et al. 2024; Khosravi et al. 2023). Claims on product labels boost the confidence of caregivers that they are making a healthy, nutritious and safer choice for their children (Dixon et al. 2024; McCann et al. 2022; Richter et al. 2022), which leads them to purchase the products. (Dixon et al. 2024; Walls et al. 2023). In addition to their influence on purchasing, claims may also be inaccurate and misleading, and/or give CPCFs a ‘health halo’ – misperception around the healthiness of food products (Her and Seo 2017) – compared to fresh alternatives. For instance, in a randomised trial conducted among parents of children aged 1–5 years in US, Hall et al. (2022) reported that exposure to a nutrition claim increased the likelihood of parents selecting a fruit drink high in added sugar instead of 100% juice or water for their children. In further examples of misleading claims, a study from the US reported parents of children 2 years of age perceiving cereals with lower than average nutritional quality as being nutritious and healthy based on product label claims related to the child's immunity and nutrient content (Harris et al. 2011). Likewise, a study by Fabiansson (2006) found a variation of up to 110% for actual versus labelled sugar content in products that claimed to be low in sugar. Other non‐nutritive claims implying standard and optimal feeding also influence parents' intentions to choose CPCFs for their children, specifically when they want to be assured of providing safe and nutritious foods for their children (Isaacs et al. 2022).

In the context of increasing availability of CPCFs of low nutritional quality, our study provides timely evidence that policymakers can use to advocate for stronger enforcement of policies regulating CPCFs. Nepal is a signatory to the World Health Assembly Resolution 69.9, which calls for ending the inappropriate promotion of foods for IYC (WHO 2017). In December 2022, Nepal adopted a mandatory food standard for cereal‐based commercial complementary food to regulate nutrient composition, promotional requirements, and food safety parameters of the cereal‐based sub‐category of CPCF (Ministry of Agriculture and Livestock Development 2023). At the time, there was no global or regional guidance, other than Codex Alimentarius standards, to refer to the nutrient composition of commercial food for IYC. Consequently, the sodium limit in the Nepal CPCF standard (0.30 g/100 g) permits considerably more sodium per 100 g than the limit of 0.04 g/100 g in SEAR NPPM. We recommend that future amendments of Nepal's CPCF standard refer to the SEAR NPPM thresholds. Our study also suggests that similar standards should be applied to ‘rusk and biscuits’ and ‘fruit and vegetable‐based products’. These standards should be easy to apply in the national context. For instance, food categories and sub‐categories, could be localised for the ease of application, by providing examples of local foods.

Regular monitoring and enforcement will be important to ensure CPCFs meet national standards, hence, it is important that relevant government officers are equipped to operationalise the standards. However, there may be political and structural barriers to operationalizing such standards. In countries like Nepal, recognised barriers to enforcement of health or food policies include industry influence, import of food products and rules over trade, competing development priorities, institutional capacity (financial and non‐financial resources) and unclarity of resources in policy documents (Amukugo et al. 2021; Fisher et al. 2021; UNICEF 2024). Our ongoing research is exploring Nepali policymaker perspectives on barriers regarding expanding and enforcing the CPCF standard. A key action for policymakers is anticipating these potential barriers and planning ahead to address and overcome them.

Future research, assessing the compliance of CPCFs available in Nepal with national or regional nutrient and labelling requirements, will give an insight into the progress made to improve the nutrient composition and labelling practices of CPCFs available for IYC. Further research is also needed to understand the use of CPCFs among Nepali parents/carers and the influence of claims on their perceptions of the healthiness of CPCFs and purchasing behaviour. Also, further research is needed to gather policymakers' perspectives on how to develop and enforce CPCF regulations in Nepal and on what additional strategies may be effective.

This study makes an important initial effort in providing empirical evidence on the nutrient composition and health information on the labels of CPCFs in Nepal using WHO SEAR NPPM. SEAR NPPM is tailored to the region and is suitable to assess both nutritional quality (aligning with Codex standards for baby foods) and promotional strategies (aligning with WHO's recommendations on ending inappropriate promotion of foods for infants and young children) of CPCFs. Another strength is the variety of stores included, particularly baby stores, which gives us confidence that we have captured most CPCF products available in the market. A limitation is that the stores were not nationally representative, but only formal stores in urban areas. This is because we wanted to capture produce diversity rather than store representativeness. Future studies are needed to explore availability, nutritional quality and promotion of CPCFs in rural Nepal. A further limitation is that we did not verify the actual nutrient content of the products in the laboratory and adopted methods of using nutrient information on the product label as used by several other studies (Bassetti et al. 2023; Coyle et al. 2024; Dunford et al. 2024; Hutchinson et al. 2021; Scully et al. 2023).

## 5 Conclusion

Sixty‐one unique CPCFs were available for purchase to Nepali. None met all the WHO SEAR NPPM energy and nutrient composition threshold, and more than half were above thresholds for sugar and sodium threshold. Few complied with SEAR NPPM labelling criteria, with some products targeting infants under 6 months of age and undermining breastfeeding. Claims that products were healthy, nutritious, and ideal for children were common on labels. With the increasing commercialisation of foods for infants and young children, the Nepalese government has an important role to play in enhancing and enforcing regulation to protect IYC and promote their healthy growth and development. Future studies assessing availability and nutritional quality of CPCFs in rural areas, and the use of CPCFs by Nepali parents/carers will enable a full understanding of the scale of the problem. Nepal has a mandatory food standard for cereal‐based commercial complementary food and ongoing monitoring will help ensure that the available CPCFs meet national standard. This study also suggests standards are needed for other CPCF's sub‐categories.

## Author Contributions

N.S. designed the study with input from C.B., P.La., P.Lo., P.K.P. N.S. conducted field work. N.S. led data extraction with support from P.K.P. N.S. led the analysis with support from C.B. and P.La. N.S. wrote the first draft of the manuscript with input from C.B. All authors critically reviewed all manuscript versions and provided their inputs. All authors read and approved the final manuscript.

## Conflicts of Interest

N.S. is supported by Deakin University Postgraduate Research Scholarship.

## Data Availability

Data is available upon request to corresponding author.
